# The bidirectional relationship between epilepsy and depression

**DOI:** 10.1192/j.eurpsy.2021.910

**Published:** 2021-08-13

**Authors:** S. Belghuith, S. Daoud, N. Smaoui, N. Farhat, S. Sakka, O. Hdiji, K.S. Moalla, M. Damak, M. Maalej Bouali, C. Mhiri

**Affiliations:** 1 Neurology, habib bourguiba hospital, sfax, Tunisia; 2 Neurological Department, habib bourguiba hospital, sfax, Tunisia; 3 Psychiatry C Department, Hedi chaker University hospital, sfax, Tunisia

**Keywords:** Epilepsy, Depression, psychiatric comorbidity

## Abstract

**Introduction:**

The relationship between epilepsy and depression has been recognized for a long time. In fact, the presence of depression could worsen the disease outcome.

**Objectives:**

we aimed to study the prevalence of depression in patients with epilepsy and to assess the determinant factors of its genesis.

**Methods:**

54 patients with epilepsy, aged more than 18 years, attending the neurology department of Habib Bourguiba Hospital, Sfax, Tunisia, were enrolled for the study. All patients were administered Mini-International Neuropsychiatric Interview (MINI) for evalution of psychiatric comorbid disorders especially depression. Socio-demographic and clinical data were collected.

**Results:**

A total of 54 patients were included, of whom 63% (n =34) were men. Mean duration of epilepsy was 20.13 years. The most frequent type of seizure was generalized 72.7%. Depression was present in 7.3 % of patients. Alcoholism (p=0.027) was significantly associated with occurrence of depression. Drug resistence (p = 0.03) and longer duration of epilepsy (p = 0.046) were significantly associated with occurrence of depression. No significant association was found between type of seizure, seizure frequency, medication compliance and depression. Depression wasn’t associated with anti-epileptic drug. We didn’t find any association between depression and other psychiatric comorbidities.

**Conclusions:**

Depression wasn’t frequent in our study contrary to literature. The possible explanations are the reduced simple size and the sensitivity of the used tool to assess depression in epilepsy. Pursuant to literature, we found significant association between Alcoholism, drug resistance and long duration of epilepsy.

